# How Big Data and Artificial Intelligence Can Help Better Manage the COVID-19 Pandemic

**DOI:** 10.3390/ijerph17093176

**Published:** 2020-05-02

**Authors:** Nicola Luigi Bragazzi, Haijiang Dai, Giovanni Damiani, Masoud Behzadifar, Mariano Martini, Jianhong Wu

**Affiliations:** 1 Laboratory for Industrial and Applied Mathematics (LIAM), Department of Mathematics and Statistics, York University, Toronto, ON M3J 1P3, Canada; daihaijiang2013@gmail.com; 2 Postgraduate School of Public Health, Department of Health Sciences (DISSAL), University of Genoa, 16132 Genoa, Italy; mariano.yy@gmail.com; 3 Department of Dermatology, Case Western Reserve University, Cleveland, OH 44195, USA; dr.giovanni.damiani@gmail.com; 4 Clinical Dermatology, I.R.C.C.S. Istituto Ortopedico Galeazzi, 20161 Milan, Italy; 5 Department of Biomedical, Surgical and Dental Sciences, University of Milan, 20122 Milan, Italy; 6 Social Determinants of Health Research Center, Lorestan University of Medical Sciences, Khorramabad 6813833946, Iran; masoudbehzadifar@gmail.com

**Keywords:** viral outbreak, public health, epidemiology, artificial intelligence, Big Data

## Abstract

SARS-CoV2 is a novel coronavirus, responsible for the COVID-19 pandemic declared by the World Health Organization. Thanks to the latest advancements in the field of molecular and computational techniques and information and communication technologies (ICTs), artificial intelligence (AI) and Big Data can help in handling the huge, unprecedented amount of data derived from public health surveillance, real-time epidemic outbreaks monitoring, trend now-casting/forecasting, regular situation briefing and updating from governmental institutions and organisms, and health facility utilization information. The present review is aimed at overviewing the potential applications of AI and Big Data in the global effort to manage the pandemic.

## 1. The Currently Ongoing COVID-19 Outbreak

From the currently available knowledge, in late December 2019, a number of cases due to an emerging virus responsible for a pneumonia of unknown cause and respiratory symptoms was observed in a cluster of patients, some of whom had visited a wet market, namely the “Huanan Seafood Wholesale Market” in the city of Wuhan, province of Hubei, located in the southern part of the People’s Republic of China [1].

The novel virus was initially termed as “2019 novel coronavirus” (2019-nCoV) and, subsequently, as “Severe Acute Respiratory Syndrome Coronavirus type 2” (SARS-CoV-2). It was first isolated on 7 January 2020. Since then, the virus has spread worldwide, reaching, as of 25 April 2020, 210 countries and infecting more than 3,000,000 patients globally, causing 200,000 deaths.

Patients infected by the virus may either be asymptomatic or symptomatic, with mild (such as fever, sore throat, and cough) to severe clinical symptoms (like pneumonia, respiratory failure and, ultimately, death) [2]. The communicable disorder caused by SARS-CoV-2 is named “coronavirus disease” (COVID-19) [3]. 

From a molecular perspective, the SARS-CoV-2 is an enveloped, single-stranded, positive-sense RNA virus and represents the eighth coronavirus that can be transmitted from human to human [4]. Bats, which are reservoir hosts of various zoonotic viruses, including the Hendra and Nipah viruses, have been indicated as putative key reservoirs of coronavirus in China [5]. From a genomic standpoint, the SARS-CoV-2 shares approximately 50% and 79% of its genetic sequence with the MERS-CoV and the SARS-CoV, respectively. Furthermore, SARS-CoV-2 shares a receptor-binding domain structure with SARS-CoV [6].

Thanks to the latest advancements in the field of computational techniques and information and communication technologies (ICTs), artificial intelligence (AI) and Big Data can help handle the huge, unprecedented amount of data derived from public health surveillance, real-time epidemic outbreaks monitoring, trend now-casting/forecasting, regular situation briefing and updating from governmental institutions and organisms, and health resources utilization information [7].

Big Data have been classically defined by three Vs: (i) velocity (in terms of the unprecedented speed of data acquisition, processing and manipulation; in this regard, Big Data are known also as “fast data”); (ii) volume (in terms of the high amount of information available); and (iii) variety (in terms of the number of the different sources and channels that can produce and release Big Data) [8,9]. 

There are various types of Big Data, based on their sources: (i) molecular Big Data (obtained by means of wet-lab techniques and OMICS-based approaches, such as genomics, and post-genomics specialties, including proteomics, and interactomics); (ii) imaging-based Big Data (like radiomics or the massive data-mining approach to extract clinically meaningful, high-dimensional information from images); (iii) sensor-based Big Data (wearable sensors); and (iv) digital and computational Big Data (with an incredible wealth of data produced by the internet, smart phones, and other mobile devices) [10,11,12,13].

In the remaining part of this paper, we will overview some of the major possible applications of AI and Big Data for the management of COVID-19. 

## 2. Short-Term Applications of Artificial Intelligence and Big Data: A Quick and Effective Pandemic Alert

Big Data can enable monitoring of the disease outbreak in real-time. With respect to previous epidemics and pandemics outbreaks, COVID-19 is unprecedented in that open-access datasets containing daily numbers of new infections broken down by country, and, in some cases, even cities, are widely available. Combined with the information we have about the movement of people, it represents the perfect dataset to combine mathematical modeling and AI.

Blue Dot, a Toronto-based start-up that uses an AI-enhanced surveillance system, seems to have been the first to detect the epidemic outbreak, several hours after its insurgence in the first reported epicenter of Wuhan, well ahead of the Chinese authorities and other international institutions and agencies [14].

Computational techniques enable us to visualize in real-time the spreading of the virus, such as the application designed at the John Hopkins University, USA. Furthermore, social Big Data, collected from social networks and other related non-conventional data streams, enable us to reconstruct early epidemiological story of the outbreak. For instance, Sun and colleagues [15] performed a population-level observational study, monitoring healthcare related websites, social networks and news reports, between 13th January and 31st January 2020, in mainland China. Authors concluded that non-classical datasets can help researchers understanding the spreading of an outbreak, in terms of health literacy, healthcare-seeking behaviors, and health resources’ utilization. Especially in the early stages of the outbreak, non-classical datasets and data streams can inform the design and implementation of effective public health measures. 

Similarly, Qin and coworkers [16] exploited Big Data to predict the number of new COVID-19 cases, either suspected or confirmed. In more detail, authors utilized a lagged series of “Social media search indexes” (SMSI) for various keywords, including COVID-19 clinical symptoms (such as dry cough, fever, chest distress, and pneumonia). Authors found that, by employing techniques such as subset selection method, new COVID-19 suspected and confirmed cases could be detected 6–9 and 10 days in advance, respectively. 

Yang and colleagues [17] utilized population migration data to populate a dynamic transmission, compartmental “Susceptible-Exposed-Infectious-Removed” (SEIR) model, combined with an AI approach, trained on the SARS data, in order to predict the COVID-19 pandemic curve. Authors demonstrated that a five-day delay in the implementation of the stringent public health measures adopted by the Chinese authorities would have resulted in an epidemic size increased by three times. Loosening/lifting the lock-down intervention would cause a second peak in the Hubei province by mid-March until late April.

Concerning another coronavirus, Song and coworkers [18] investigated online diffusion of information, spread of fear, and perceived risk of contracting MERS infection during the MERS outbreak in South Korea in May-June of 2015. Buzz conveying negative feelings was found to be more prevalent in online discussion boards, Twitter, and online cafes than news sites and blogs. News buzz, but not rumor buzz, correlated with positive emotions and the mention of taking immunity-boosting food. 

However, a particular aspect to which public health authorities should pay attention is another V of the three Vs of Big Data: V of veracity, that is to say, the accuracy and reliability of the data collected. The processing and modeling of Big Data should incorporate such uncertainty and deal with it, ensuring robustness of the findings. To date, this is still an open challenge. 

## 3. Short-Term Applications of Artificial Intelligence and Big Data: Tracking and Diagnosing COVID-19 Cases

Having a reliable, sensitive and specific diagnostic test is of paramount importance in the prevention and control of infectious disorders. Researchers have succeeded in establishing a molecular kit able to quickly and accurately capture the proper diagnosis and distinguish between COVID-19 and SARS-CoV. Salivary diagnostics seems to hold great promise in effectively detecting the virus. Together with molecular assays and tests, ether multiplex nucleic acid amplification or microarray-based, high-resolution CT of the chest is fundamental for monitoring the disease course and its evolution in terms of severity and response to treatment. Currently ongoing research is also trying to identify early radiological predictors of prognosis, which would be extremely helpful in stratifying patients with COVID-19 and in their clinical management [7,14,19]. 

AI can facilitate the diagnosis of COVID-19 cases. For instance, Infervision is a start-up that employs deep learning medical imaging platforms for facilitating quick diagnosis of COVID-19 cases via the recognition of specific lung features [7,14].

Furthermore, block-chain technology is a unique decentralized system of recording, verifying and approving data and carrying out a series of transactions. It is characterized by a high level of security and enables the delivery of patient-centered healthcare services, enhanced public health surveillance, management of outbreaks and a quick and effective decision-making process [20,21,22]. A low-cost block-chain and AI-coupled self-testing and tracking system has been proposed for managing the COVID-19 pandemics, in developed settings (to avoid overwhelming and straining public health capacity and healthcare/laboratory infrastructure) and in developing, resource-limited contexts [20]. 

## 4. Medium-Term Applications of Artificial Intelligence and Big Data: Identifying a Potential Pharmacological Treatment

Currently, no officially approved therapeutic options exist for the treatment of COVID-19. Physicians should provide patients with supportive management and screen their nutritional status before administering any drugs. 

Due to the emergency situation, since it would be time- and resource-consuming to develop ad hoc specific therapeutics, scientists are exploring three major approaches: (i) the feasibility of utilizing already existing broad-spectrum anti-viral drugs, (ii) the possibility of modifying/adapting them, taking into account the biochemical and biophysical features of the novel virus, and (iii) exploiting available pharmaceutics (either belonging to Western or Chinese traditional medicine) for other therapeutic purposes (drug repositioning or repurposing) [2]. 

These strategies are aimed at targeting the virus by (i) interfering directly with it, or by (ii) blocking/inhibiting its related biological process and events (such as viral entry and replication), or targeting human immune system, by (iii) enhancing it [2]. Advancements in the genomic and post-genomic fields enable to quickly scrutinize genome sequences, looking for similarity with other genomes and discovering potential druggable targets. 

Candidate drugs against COVID-19 include neuraminidase inhibitors such as Oseltamivir and Peramivir, anti-retroviral protease inhibitors like Ritonavir/Lopinavir, nucleotide analogs as Remdesivir, nucleoside inhibitors as Ribavirin, influenza hemagglutinin-mediated fusion inhibitors like Arbidol/Umifenovir and anti-malarials as Chloroquine, among others. 

Most patients with COVID-19 are being treated with Oseltamivir, despite some recent Cochrane reviews questioning its effectiveness in the management of influenza. Oseltamivir has been shown to result in small clinically relevant effects, with a remarkable occurrence of side effects. Data concerning Peramivir are even more limited, with few randomized clinical trials (RCTs) available, thus hindering a comparison between the two neuraminidase inhibitors. Moreover, in-vitro testing assays have failed to show the inhibition of cytopathic effects of SARS-CoV [2], demonstrating, on the contrary, the efficacy of some commercially available interferons. However, to date, these have been less utilized for the clinical management of patients with COVID-19. 

The usage of Ribavirin, a pro-drug of ribavirin triphosphate and a competitive inhibitor of the enzyme inosine monophosphate dehydrogenase, which is crucial for the biosynthesis of guanosine and nucleic acids synthesis, is neither supported by cellular nor clinical data, with several studies reporting, instead, contrasting findings and clinically relevant toxicities that may result in discontinuation of therapy [2]. For Arbidol, limited data exist [2] and generally its use in treating severe acute respiratory infections is not recommended. In SARS patients, Lopinavir/Ritonavir, besides exerting in-vitro anti-viral activities, has contributed to sparing steroid doses, reducing hospitalizations and nosocomial infections, resulting in a decreased viral load and increased peripheral lymphocyte count [2]. 

Few patients are being treated with gancivlovir, an acyclovir analogue and inhibitor of the Herpesvirus family including cytomegalovirus (CMV). Ganciclovir is approved for the treatment of complications arising from AIDS-associated CMV infection, but in-vitro assays have not shown any inhibition of the cytopathic effects of SARS-CoV [2]. 

Furthermore, some physicians administer corticosteroids, the evidence of which in terms of clinical outcomes and complications/death prevention is moderate according to the Cochrane reviews. In a systematic review of the treatment effects of candidate drugs against SARS-CoV, 25 out of 29 studies assessed mentioning steroid, its use was found to be inconclusive, with four of them showing potential harm to patients’ health [23]. Similarly, the effectiveness of antibiotics in the treatment of viral pneumonia appears to be limited [2]. 

In conclusion, an effective treatment for COVID-19 is still lacking.

AI can help quickly identify potential therapeutics and candidate vaccines. For instance, a collaboration between an AI start-up, BenevolentAI, and a university institution, the Imperial College London, UK, has led to the discovery that baricitinib, a biologic used for the treatment of moderately to severely active rheumatoid arthritis in adults, inhibitor of the janus kinases JAK1 and JAK2, may exert anti-viral effects [24], whereas another start-up, Insilico Medicine, located in Hong Kong, has reported to have found six new drugs that may inhibit viral replication [14].

Concerning traditional Chinese medicine, by employing network pharmacology analysis, Zhang and coauthors [25] systematically screened natural compounds used in Chinese treatment, and found 13 of them to exert potential anti-COVID-19 effects in terms of the regulation of viral replication, modulation of immune and inflammatory pathways and hypoxia cascade.

## 5. Medium-Term Applications of Artificial Intelligence and Big Data: Facilitating the Implementation of Public Health Interventions

During an outbreak, resources are limited and can be quickly consumed. As such, to avoid wasting resources and to better allocate those available, the Chinese government has supplemented classical data collection methods with sophisticated computational systems and advanced techniques that have helped identify at-risk subjects.

A start-up, Megvii, has announced the development of sophisticated body and face detection and dual sensing via infrared cameras and visible light, as thermal scanners for rapidly screening subjects transiting in a crowded place and identifying fever and high temperature, potentially related to COVID-19.

Ant Financial Services Group, formerly known as Alipay, of the Alibaba group, have devised AI-based applications, based on parameters like self-reported health status, history of travels and contacts, that can identify COVID-19 cases. This system has been implemented in the county of Shizhu, located in Chongqing, in the southwestern part of the People’s Republic of China, to monitor the flow of people during the Chinese Lunar New Year holiday, which has favored the spreading of the virus. Based on this tool, it has been possible to inform the measure of quarantine. Similarly, in the provinces of Zhejiang, Sichuan and Hainan, advanced data analyses have been exploited to reconstruct social interactions and perform contact tracing. 

Srinivasa Rao and Vazquez [26] have described a framework based on an AI algorithm that would enable a quick identification of infected cases, performing a risk assessment and evaluation according to the symptoms and signs related to the novel coronavirus. This would be done via a web- or mobile-based survey. Further, depending on the replies, the algorithm is able to send alerts to clinics or mobile health units, for health visits and confirmation of the case. 

## 6. Long-Term Applications of Artificial Intelligence and Big Data: Building Smart, Health, Resilient Cities

In recent years, profound societal phenomena, including globalization and rapid urbanization together with population explosion and demographic changes, have deeply modified our lifestyles. Approximately more than half of humanity dwell in urban areas and settlements, with this figure being expected to significantly increase up to two-thirds of the global population by 2050. In the Western countries, whereas, at the beginning of the 20th century, less than half of the population lived in urban areas, this percentage had doubled at the beginning of the new century and it can be anticipated that it will further rise in the coming decades. 

“Make cities and human settlements inclusive, safe, resilient and sustainable”, was ambitiously declared by the eleventh Sustainable Development Goal (SDG-11) of the United Nations, and has been reaffirmed during the 2016 United Nations Habitat III on housing and sustainable urban development in Ecuador (the “New Urban Agenda”). It is becoming more and more imperative. Environmental (pollution, waste disposal, climate change) and health (communicable diseases, such as food- or vector-borne infections) challenges represent serious concerns that may threaten citizens’ livability and quality of life [27].

All this requires a multi-disciplinary approach to be properly and effectively addressed by all the stakeholders (urban designers and planners, workers in the field of public health), also incorporating citizens in the planning of a sustainable urban management and empowering them. A highly integrated systems approach is of crucial importance, in that a healthy city is like a living, dynamic organism. Smart interconnected devices, wearable sensors and other innovative technologies like smart phones, telecommunications, networks and GPS, enable to collect an incredible wealth of data in real time that are user-centered and technology-driven, allowing: (1) quality monitoring, transparency and accountability, (2) resource allocation optimization, (3) citizen participation and inclusion, and 4) resilience and adaptation to exogenous events. 

In recent years, several cities and communities in Spain, New Zealand, United States, United Kingdom, Canada and United Arab Emirates have launched initiatives and challenges with the aim of promoting research and innovation in the field. 

According to a recent systematic review of the literature, smart cities can enable population-wide surveillance, counteract ageing, by promoting active ageing, socialization and healthy lifestyles, include disabled and marginalized people, and provide quick and effective responses to emergencies and disasters such as outbreaks [27].

However, despite the importance of such a topic, there is a dearth of mathematical models, as stated by Grindrod and colleagues [28]. The field of “mathematics of smart cities” is particularly challenging in that modeling communities is not a trivial task in terms of city boundaries and their relationship with the surrounding environments. Capturing the time-dependency of interactions among the different components of a smart city is rather demanding. 

In the aftermath of disasters, the risk of communicable disorders is high. Designing and implementing policies for properly managing disasters and emergencies should be holistic and multi-level, incorporating prevention, defense, mitigation, preparation, response and recovery. Current policies suffer from gaps, in that, instead of being multi-criteria, broader and highly integrated, foreseeing future events based on risk-assessment scenarios and planning in advance, focus mainly on disaster resistance and are based on contingent evaluations based on administrative and political pressures. 

AI and Big Data can provide local decision- and policy-makers with informed, evidence-based predictions. Allam and Jones [29] have investigated the COVID-19 outbreak adopting an urban standpoint, showing how smart cities and smart networks can exploit high-quality, enhanced standardized protocols for data sharing during emergencies, to better manage such situations.

However, differently from the previous sections, this section still remains highly speculative, with applications currently being experimental. 

## 7. Artificial Intelligence and Big Data for COVID-19: Conclusions and Future Prospects

The potential applications of AI and Big Data for the management of COVID-19 are summarized in Table 1.

AI and Big Data appear to have enormous potential for the management of COVID-19 and other emergencies, and their role is anticipated to increase in the future. AI and Big Data can be used to track the spread of the virus in real time, and plan and lift public health interventions accordingly, monitor their effectiveness, repurpose old compounds and discover new drugs, as well as identify potential vaccine candidates and enhance the response of communities and territories to the ongoing pandemic. These emerging approaches can be exploited together with classical surveillance: whilst the latter enables data analysis and interpretation, the former uncovers hidden trends and patterns, which can be used to build predictive models. 

As maintained by Hua and Shaw [30], despite the initial delay from Chinese authorities in responding to the outbreak, “a unique combination of strong governance, strict regulation, strong community vigilance and citizen participation, and wise use of big data and digital technologies” have been among the major factors of the success of the People’s Republic of China in the efforts of containing COVID-19. Further research is warranted to explore how to apply such sophisticated technologies preserving human rights and privacy, whilst, at the same time, ensuring high standards. 

## Figures and Tables

**Table 1:** Possible applications of Artificial Intelligence and Big Data for the management of the ongoing COVID-19 outbreak.

Time-Scale	Possible Application	Example
Short-term (weeks)	Rapid identification of an ongoing outbreak	AI can facilitate real-time epidemiological data collection, risk-assessment, decision-making processes, and design/implementation of public health interventions
	Diagnosis and prognosis of COVID-19 cases	Recognition of specific diagnostic and prognostic features
Medium-term (months)	Identification of a potential therapeutic option	Identify already existing drugs/discovering new molecules
Long-term (decades)	Enhancing cities and favoring the development of healthy, smart, resilient cites	Design new standardized protocols for sharing data and information during emergencies
